# Endothelial Cell-Specific Molecule 1 Promotes Endothelial to Mesenchymal Transition in Renal Fibrosis

**DOI:** 10.3390/toxins12080506

**Published:** 2020-08-06

**Authors:** Tung-Wei Hung, Chao-Yang Chu, Chen-Lin Yu, Chu-Che Lee, Li-Sung Hsu, Yong-Syuan Chen, Yi-Hsien Hsieh, Jen-Pi Tsai

**Affiliations:** 1 Division of Nephrology, Department of Medicine, Chung Shan Medical University Hospital, Taichung 40201, Taiwan; a6152000@ms34.hinet.net; 2 School of Medicine, Chung Shan Medical University, Taichung 40201, Taiwan; ritahero318@gmail.com; 3 Institute of Medicine, Chung Shan Medical University, Taichung 40201, Taiwan; s0303019@gm.csmu.edu.tw (C.-L.Y.); lsh316@csmu.edu.tw (L.-S.H.); kevin810647@gmail.com (Y.-S.C.); 4 Department of Medicine Research, Buddhist Dalin Tzu Chi Hospital, Chiayi 62247, Taiwan; turtle12062001@yahoo.com.tw; 5 Department of Biochemistry, School of Medicine, Chung Shan Medical University, Taichung 40201, Taiwan; 6 Clinical Laboratory, Chung Shan Medical University Hospital, Taichung 40201, Taiwan; 7 School of Medicine, Tzu Chi University, Hualien 97010, Taiwan; 8 Division of Nephrology, Department of Internal Medicine, Dalin Tzu Chi Hospital, Buddhist Tzu Chi Medical Foundation, Chiayi 62247, Taiwan

**Keywords:** endothelial-to-mesenchymal transition, renal fibrosis, endothelial cell specific molecule 1, motility, migration, unilateral ureter obstruction

## Abstract

The endothelial-to-mesenchymal transition (EndoMT) is involved in the complex pathogenesis of renal fibrosis. The soluble proteoglycan endothelial cell-specific molecule 1 (ESM1) is significantly upregulated in many tumor cells and cirrhosis-related disease. The role of ESM1 in renal fibrosis is unknown. This study investigates the role of ESM1 in renal fibrosis, using an in vivo unilateral ureteral obstruction (UUO) mouse model of renal fibrosis and in vitro mouse kidney MES 13 cells overexpressing ESM1. We observed that ESM1 overexpression significantly increased the motility and migration of MES 13 cells, independent of cell viability. In ESM1-overexpressing MES 13 cells, we also observed elevated expression of mesenchymal markers (N-cadherin, vimentin, matrix metallopeptidase 9 (MMP9)) and the fibrosis marker α-smooth muscle actin (α-SMA) and decreased expression of the endothelial marker vascular endothelial cadherin (VE-cadherin) and CD31. In a mouse model of fibrosis induced by unilateral ureter obstruction, we observed time-dependent increases in ESM1, α-SMA, and vimentin expression and renal interstitial collagen fibers in kidney tissue samples. These results suggest that ESM1 may serve as an EndoMT marker of renal fibrosis progression.

## 1. Introduction

Chronic kidney disease (CKD), characterized by progressive renal dysfunction, is recognized as a major public health problem with high morbidity and mortality worldwide [1]. Renal fibrosis is common in the progression of CKD of all etiologies. Renal fibrosis involves the excessive deposition of extracellular matrix in glomeruli and interstitium resulting from interactions between a variety of cells and cytokines [2]. The pathologic findings of renal fibrosis include glomerulosclerosis, tubulointerstitial fibrosis, inflammatory infiltration, and the loss of renal parenchyma characterized by tubular atrophy, capillary loss, and podocyte depletion [3,4,5].

Endothelial cell-specific molecule 1 (ESM1), also known as endocan, is a 50-kDa soluble proteoglycan (PG) secreted by vascular endothelial cells [6]. ESM1 is mainly synthesized by endothelial cells in the lung and kidney, where it binds to hepatocyte growth factor/scatter factor through its glycan moiety [7]. As ESM1 expression is particularly high in inflamed endothelium, ESM1 is thought to play a role in the pathogenesis of vascular disorders, inflammation, and endothelial dysfunction [8]. Plasma ESM1 levels correlate with inflammation severity and poor survival in coronary artery disease [9], chronic kidney disease [10], IgA nephropathy [11], and sepsis [12]. Further, ESM1 expression is elevated in patients with systemic sclerosis [13,14]. In kidney transplantations, plasma and urinary ESM1 levels may serve as potential markers of microvascular inflammation and are elevated in patients with antibody-mediated rejection [15]. Recent studies have shown that injury to glomerular endothelial cells directly contributes to podocyte and mesangial cell damage and the development of glomerulosclerosis [16,17,18].

The endothelial-to-mesenchymal transition (EndoMT) is a complex process by which certain endothelial cell subsets lose endothelial characteristics and transform into mesenchymal or smooth muscle cells [19]. Li and Bertram [20] reported that the EndoMT is a novel pathway in the development of fibrosis in diabetic nephropathy and other animal models of kidney fibrosis. Zeisberg et al. [21] examined the contribution of the EndoMT to the development of renal fibrosis in three different animal models of end-stage kidney disease: unilateral ureteral obstruction, streptozotocin-induced diabetic nephropathy, and a COL4A3 knockout murine model of Alport’s syndrome. To investigate the role of ESM1 in the progression of renal fibrosis, this study aims to determine whether ESM1 overexpression promotes renal fibrosis and EndoMT pathways using in vitro mouse kidney cell culture and an in vivo mouse model of renal fibrosis induced by unilateral ureteral obstruction.

## 2. Results

### 2.1. Upregulation of ESM1 in Unilateral Ureteral Obstruction (UUO)

**Figure 1:**
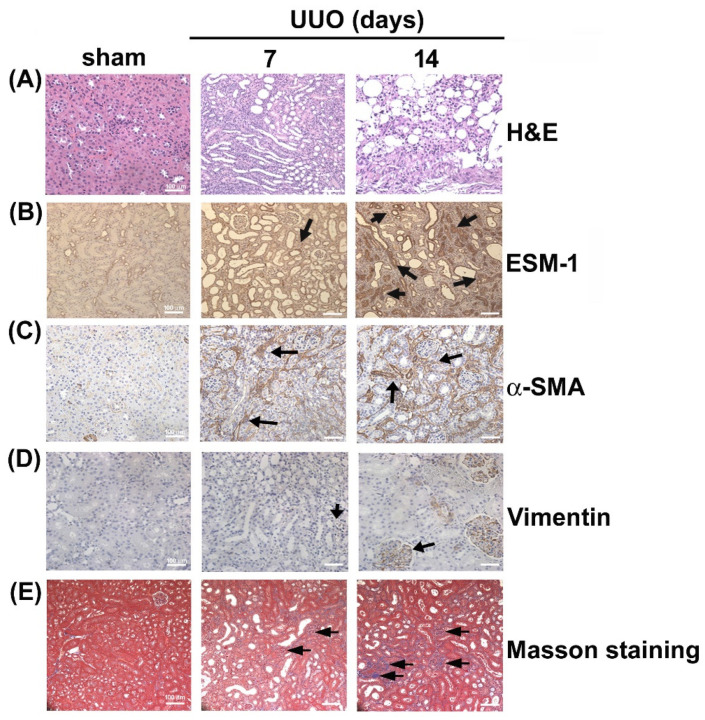
Unilateral ureteral obstruction (UUO) induces renal fibrosis and upregulates endothelial cell-specific molecule 1 (ESM1) expression in mice. Male C57BL/6 mice were subjected to UUO surgery and sacrificed 7 or 14 days post-surgery. (**A**) Morphological features of kidney tissue samples visualized by hematoxylin and eosin (HE) staining. (**B**–**D**) Expression of ESM1, α-smooth muscle actin (α-SMA), and vimentin as detected by immunohistochemical staining. (**E**) Interstitial collagen fibers in renal tissues visualized by Masson’s Trichrome staining. Scale bar = 50 µm.

Morphological features of hematoxylin and eosin (HE) stained kidney tissue sections were compared between the UUO and sham surgery groups (Figure 1A). Immunohistochemical assays showed that in the UUO group, the expression of ESM1 increased markedly (day 7 and 14) (Figure 1B), and α-SMA and vimentin expression also increased significantly, in a time-dependent manner, compared to the sham surgery group (Figure 1C,D). Masson tissue staining revealed a time-dependent increase in blue-stained interstitial collagen fibers in the UUO group, but no such fibers in the sham surgery group (Figure 1E). These results suggest that ESM1 may be involved in renal fibrosis progression.

### 2.2. Overexpression of ESM1 in Mouse MES 13 Cells

**Figure 2:**
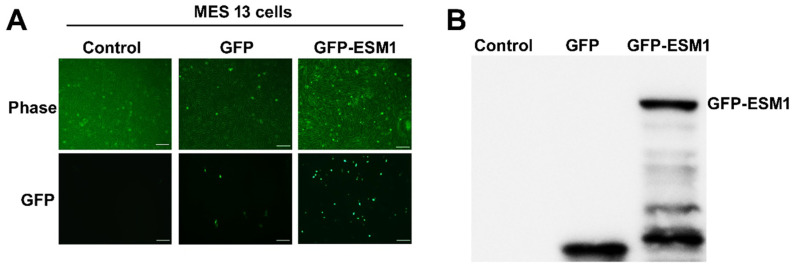
Overexpression of ESM1 in MES 13 cells. (**A**) After the transfection of GFP and GFP-ESM1 plasmids into MES 13 cells, GFP-ESM1 expression was observed by fluorescence microscopy. (**B**) ESM1 protein expression levels were assessed by immunoblot analysis. Control, untransfected cells. Data are expressed as the mean ± SE of three independent experiments. Scale bar = 20 µm.

To elucidate the biological function of ESM1 in the progression of renal fibrosis, ESM1 was overexpressed in MES 13 cells. GFP-ESM1 protein expression in the transfected cells was confirmed by fluorescence microscopy (Figure 2A). Immunoblotting also confirmed the ectopic expression of ESM1 in MES 13 cells (Figure 2B).

### 2.3. Overexpression of ESM1 Had No Effect on the Growth of MES 13 Cells 

**Figure 3:**
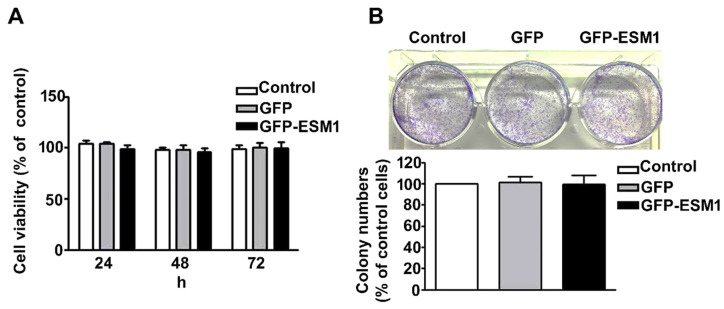
Overexpression of ESM1 has no effect on the growth of MES 13 cells. (**A**) viability of control, GFP and GFP-ESM1—expressing MES 13 cells as determined by the MTT assay. Data are presented as mean ± standard error for the three replicates. (**B**). Representative images of the colony formation potential of MES 13 cells. Data are expressed as the mean ± SE of three independent experiments.

To investigate the effect of ESM1 overexpression on the growth of MES 13 cells, transfected cells were analyzed by the MTT assay and the colony-forming assay. We found that overexpression of ESM1 did not alter the short-term (Figure 3A) or long-term (Figure 3B) growth of MES 13 cells. These results indicate that ESM1 overexpression does not affect the viability of MES 13 cells.

### 2.4. Overexpression of ESM1 Increased Motility and Migration of MES 13 Cells 

**Figure 4:**
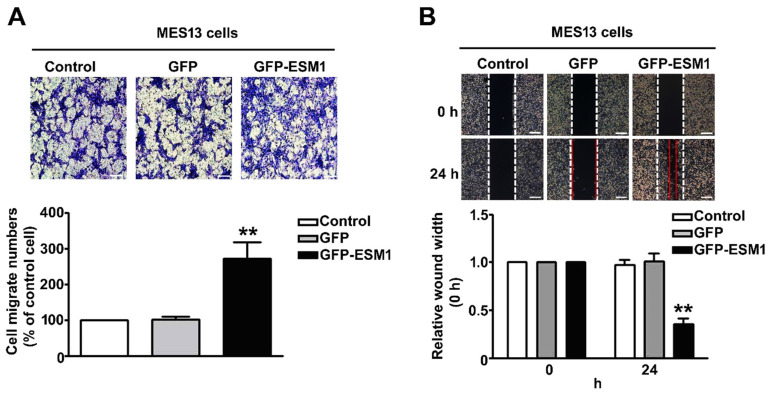
Overexpression of ESM1 increased the motility and migration of MES 13 cells. (**A**) Control, GFP or GFP-ESM1 expressing MES 13 cells (2 × 10^5^/well) were seeded in the top chamber for 24 h by the Boyden chamber migration assay. Cells that migrated through the membrane were stained and quantified. Scale bar = 50 µm (**B**) Wound healing assays were used to evaluate the motility of control, GFP and GFP-ESM1 expressing MES 13 cells. ** *p* < 0.01 vs. control cells. Control; untransfected cells. Data are expressed as the mean ± SE of three independent experiments. Scale bar = 100 µm.

To determine whether overexpression of ESM1 regulates the migration and motility of MES 13 cells, transfected cells were assessed through the in vitro Boyden chamber assay and the wound healing assay. Our data indicated that overexpression of ESM1 significantly increased the cell migration (Figure 4A) and motility (Figure 4B) of MES 13 cells, compared with control cells. These results suggest that ESM1 promotes cell motility and migration of MES 13 cells.

### 2.5. Overexpression of ESM1 Induced EndoMT in MES 13 Cells

**Figure 5:**
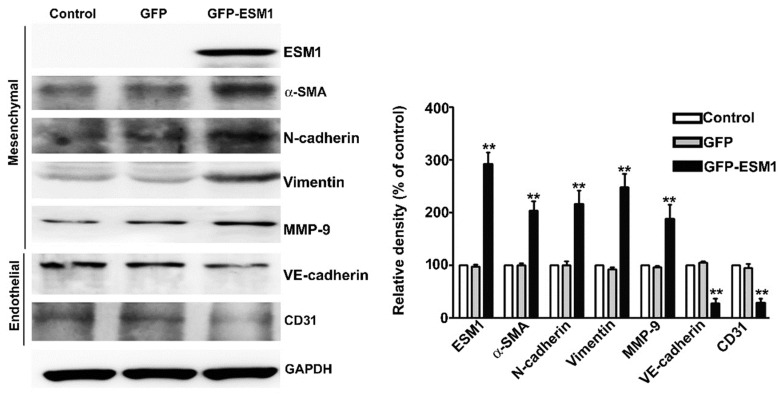
Overexpression of ESM1 results in changes in endothelial-to-mesenchymal transition (EndoMT) marker expression in MES 13 cells. Protein expression levels of ESM1, N-cadherin, vimentin, α-SMA, MMP9, vascular endothelial cadherin (VE-cadherin) and CD31 in control, GFP and GFP-ESM1 expressing MES 13 cells were assessed by immunoblot analysis. GAPDH served as an internal control. Relative quantitative results are shown in the bar graph. ** *p* < 0.01 vs. control cells. Data are expressed as the mean ± SE of three independent experiments.

To investigate the molecular mechanism of overexpression of ESM1 increased the migration of MES 13 cells, immunoblotting was used to examine the expression of EndoMT-related proteins. We found that ESM1 overexpression resulted in the increased expression of N-cadherin, MMP-9, vimentin and α-smooth muscle actin (α-SMA) and decreased the expression of vascular endothelial cadherin (VE-cadherin) and CD31 in MES 13 cells, compared with control cells (Figure 5). These observations indicate that ESM1 may play an important role in the progression of kidney fibrosis through EndoMT regulation.

## 3. Discussion

**Figure 6:**
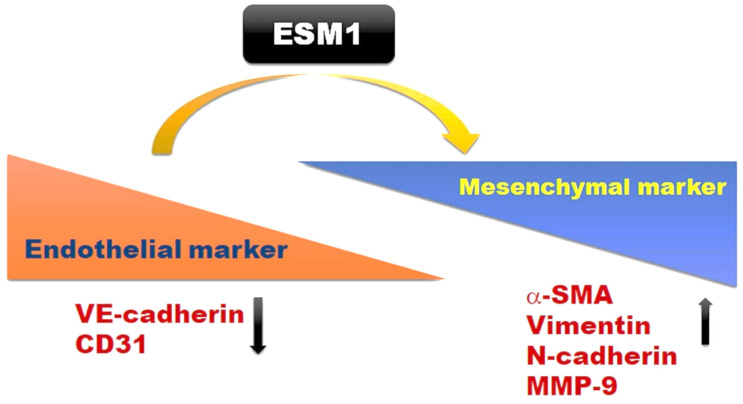
Graphical representation of the proposed molecular mechanism of ESM1-mediated EndoMT in renal fibrosis. In MES 13 cells, overexpression of ESM1 results in increased expression of mesenchymal markers (N-cadherin, vimentin, matrix metallopeptidase 9 (MMP9) and the fibrotic marker α-SMA and decreased expression of the endothelial markers VE-cadherin and CD31, accompanied by renal fibrosis.

The current study investigates the role of ESM1 in renal fibrosis in vivo and in vitro. In UUO mice, we observed increased interstitial fibrosis, collagen fibers, and ESM1 expression in vivo. In in vitro cell culture of mouse mesangial MES 13 cells, ESM1 overexpression significantly induced fibroblast formation and interstitial fibrosis (Figure 6). These results suggest that ESM1 may be an EndoMT marker of renal fibrosis.

Accumulating evidence suggests that ESM1 has prognostic value in pathological conditions including cancer, sepsis, inflammatory disorders, hypertension, transplant rejection, and chronic kidney disease [22,23,24]. Su et al. observed that serum ESM1 levels may reflect the degree of chronic renal allograft injury, and there in vitro study showed that tumor necrosis factor-α (TNF-α) induces high expression of ESM1 and Transforming growth factor-beta 1 (TGF-β1) in human umbilical vein endothelial cells [25]. Samouilidou et al. found that a high level of serum ESM1 was positively associated with low density lipoprotein cholesterol (LDL-C), total cholesterol, and downregulation of paraoxonase (PON) in CKD patients [26]. In non-dialysis chronic kidney disease patients (stage 5), circadian heart rate variability correlates positively with serum ESM1 concentration [27]. Toshikuni et al. showed that liver cirrhosis patients have high levels of serum ESM1 and that this protein may be a survival marker of liver cirrhosis [28] and cirrhotic cardiomyopathy [29]. ESM1 overexpression was reported to increase the expression of tumor necrosis factor-α (TNF-α) and monocyte chemotactic protein-1, which are involved in liver inflammation and fibrosis [30]. These observations suggest that ESM1 may be involved in the progression of several diseases involving fibrosis. Previous studies have suggested that ESM1 is secreted by vascular endothelial cells in several organs, but no direct evidence has shown that ESM1 is involved in the molecular mechanism underlying renal fibrosis. Our results from HE staining, Masson staining, and immunohistochemically analysis confirm that ESM1 expression is associated with fibrotic and mesenchymal changes in the kidneys of UUO-treated mice. However, the clinical significance of ESM1 in CKD patients is not clear, and further research is needed to determine whether serum ESM1 levels are of diagnostic value for patients with CKD.

The EndoMT is known to be involved in the generation of myofibroblasts in fibrosis and has been suggested to promote fibrosis [21]. During the EndoMT, endothelial cells lose their adhesion and apical-basal polarity to form highly invasive, migratory, spindle-shaped, elongated mesenchymal cells [31]. Evidence indicates the involvement of EndoMT in multiple fibrotic diseases in animal models of cardiac, pulmonary, and renal fibrosis [21,32,33] and in human tissue fibrosis [34,35]. The EndoMT is characterized by the downregulation of endothelial markers, such as VE-cadherin and CD31, and the upregulation of mesenchymal markers such as N-cadherin, α-SMA, vimentin, and MMP-9 [33]. Recent reports have shown that ESM1 is associated with endothelial dysfunction, hypertension, kidney transplantation [36], and renal transplant rejection [37]. Further investigation is required to elucidate the molecular mechanism by which ESM1 promotes the EndoMT and cell migration and its contribution to the progression of renal fibrosis. This study presents the first evidence to suggest that elevated ESM1 expression promotes renal fibrosis by inducing the EndoMT. Therefore, ESM1 may play an important role in the progression of chronic kidney disease and may serve as a marker for the clinical diagnosis of renal fibrosis. It is noteworthy that ESM1 may be a molecular target of renal fibrosis, and some clinical trials are proving that antifibrotic therapies are obviously worth clinical application in the future.

Emerging evidence has shown that TGF-β induces EndoMT of kidney interstitial fibrosis through the TGF-β/Smad and Akt/mTOR/p70S6K signalling pathways [38]. Other reports have shown TGFβ-induced mesenchymal (vimentin and fibronectin) and inhibition of endothelial genes (VE-Cadherin) through activation of SMAD2/3 [39]. However, TGF-β-induced activation of Erk1/2 and p38 MAPK inhibits VE-cadherin expression and activates the Snail which induces mesenchymal differentiation in noncanonical pathways [40,41]. Based on these observations—which indicate the pivotal role of the TGF-β regulated EndoMT pathway in renal fibrosis—we will focus on the molecular mechanism of ESM1-induced EndoMT procedure of renal fibrosis, predicting the possibility for ESM1 as an up- or downstream target protein of TGF-β/Smad or MAPKs signaling pathway, and ESM1 could be an important target for renal fibrosis therapies in the future.

## 4. Conclusions

This study proposes the overexpression of ESM1 promotes cell migration and renal fibrosis progression by regulating the EndoMT pathways in vitro and in vivo.

## 5. Materials and Methods

### 5.1. Chemical Reagent 

Dimethyl sulfoxide(DMSO), MTT and crystal violet were purchased from Sigma (St. Louis, MO, USA). Cell Signaling Technology Inc. (Beverly, MA, USA). Lipofectamine 3000 reagents were purchased from Thermo Fisher Scientific (Waltham, MA, USA). The minimum essential media (MEM) was obtained from Gibco-Invitrogen Corporation (Gibco, Carlsbad, CA, USA). Fetal bovine serum (FBS), penicillin-streptomycin solution, and trypsin were purchased from HyClone (Logan, UT, USA). Antibody against ESM1 (abcom, ab56914), N-cadherin (iReal Biotechnology Co; IR46-143), vimentin (BioVision, 3634-100), α-SMA (Santa Cruz, sc-53015), GFP (Cell Signaling, 2956S), matrix metallopeptidase 9 (MMP9) (ABclonal, A0289), VE-cadherin (Santa Cruz, sc-9989), CD31 (Santa Cruz, sc-376764).

### 5.2. Cell Culture

Mouse renal glomerular mesangial cells (MES 13) were provided by Chien-Chun Li (Department of Nutrition, Chung Shan Medical University) and cultured in Dulbecco’s modified Eagle medium (DMEM)/Nutrient Mixture F-12 supplemented with 1× penicillin-streptomycin and 10% fetal bovine serum (FBS) in a 5% CO_2_ incubator at 37 °C. 

### 5.3. Gene Transfection

The GFP-ESM1 plasmid was purchased from GENEWIZ (Takeley, UK) and the ESM1 sequences as previously reported [42]. For transfection, 3 μg GFP or GFP-ESM1 plasmid in Opti-MEMTM medium (Thermo Fisher, Waltham, MA, USA) was mixed with 6 µL of Lipofectamine 3000 reagent (Thermo Fisher Scientific, Waltham, MA USA) for 20 min, then added to DMEM/F12 medium (10% FBS) and incubated with MES 13 cells for 48 h. The GFP transfection efficiency was determined by fluorescence microscopy. Cells were assessed for the protein expression of ESM1 and EndoMT markers by Western blot analysis.

### 5.4. Cell Viability Assay

The cell viability assay measured the cell growth, as previously reported [43]. GFP or GFP-ESM1 plasmid was transfected into MES 13 cells for 24, 48, or 72 h, followed by a 4-h incubation in 1 mL MTT reagent (0.5 mg/mL). The resulting formazan precipitate was dissolved in isopropanol reagent for 15 min and quantified by enzyme-linked immunosorbent assay (absorption wavelength, 570 nm). The quantity of the formazan product is directly proportional to the number of viable cells in the culture medium.

### 5.5. Cell Proliferation Assay

The colony formation assay was used to determine cell proliferation, as previously reported [44]. Control, GFP or GFP-ESM1 cells (1 × 10^4^/well) were seeded into 6-well culture plates and incubated for 7 days in MEM culture medium. After 7 days, the cells were fixed with 4% paraformaldehyde for 10 min and stained with 0.1% crystal violet for 15 min. Visible colonies were counted under a microscope.

### 5.6. Wound Healing Assay

Transfected cells were seeded in 6-well plates (2 × 10^5^ cells/well) and incubated until they reached 90% confluence. A 200-µL pipette tip was used to scratch a wound into each well, followed by incubation with the proliferation inhibitor mitomycin c (2 μg/mL) in serum free MEM medium for 24 h. The suspended cells were washed and removed in a serum-free medium. Cell migration was assessed at 0, 12, and 24 h using an inverted microscope. The average cell motility rate was calculated as the relative width of the wound divided by time.

### 5.7. Cell Migration Assay

The cell migration assay was performed by the 24-well Boyden chamber analysis, as previously reported [45]. Briefly, control, GFP or GFP-ESM1 MES 13 cells (4 × 10^5^/well) were seeded in a 6 cm dish for 24 h. Control, GFP- or GFP-ESM1 MES 13 cells (1 × 10^5^/well) was added to the upper chamber. The lower chamber had 35 μL 10% FBS containing MEM medium added. The chambers are divided by an uncoated membrane with 8 µm diameter polycarbonate membranes. After 24 h, the polycarbonate membranes were removed and fixed with methanol, then stained with crystal violet, and the number of cells was counted visually in four random fields under a light microscope.

### 5.8. Western Blot Analysis

Western blotting was performed as previously reported [46]. Cells were lysed using NETN buffer (20 mM Tris; pH 8.0, 1mM EDTA; pH 8.0, 150 mM NaCl, and 0.5% NP-40) and cocktail (Pierce Biotechnology, Rockford, IL, USA). Proteins were quantified using the Braford assay, separated by 10–12% SDS-PAGE, and transferred to a polyvinylidene difluoride (PVDF) membrane for 1 h. The membrane was then incubated in 5% non-fat milk dissolved in TBST (1X Tris-Buffered Saline, 0.1% Tween 20) buffer for 2 h at room temperature, followed by an overnight incubation with the primary antibody at 4 °C. After incubation with the secondary antibody, proteins were quantified using the ECL detection reagent (Millipore, Billerica, MA, USA) and a LAS 4000 mini luminescent image analyzer.

### 5.9. UUO Mouse Model

The previously reported UUO mouse model was used for these experiments [47]. These studies were reviewed and approved by the Institutional Animal Care and Use Committee (IACUC) of Chung Shun Medical University (IACUC:2316). Male C57BL/6 (6 weeks) mice were purchased from the National Laboratory Animal Center, Taipei City, Taiwan (Date: 18 February 2020). Mice were randomly assigned to three groups (*n* = 5 per group): sham surgery, UUO-7 days, and UUO-14 days. Renal fibrosis progression was measured at 0, 7, and 14 days after surgery after sacrificing the mice and collecting the left kidneys for analysis. For morphology analysis, kidney tissues were fixed with formaldehyde and embedded in paraffin, and then cut into 3 μm sections and placed on slides. These slides were deparaffinized twice in xylene and rehydrated in gradient ethanol, and then stained with hematoxylin-eosin reagents.

### 5.10. Immunohistochemical Analysis and Masson’s Trichrome Staining

The immunohistochemical assay was performed with the Rapid Science company (Taichung, Taiwan). The tissues mounted on glass slides were stained with primary antibodies against anti-ESM1, anti-Vimentin, and anti-α-SMA in 1∶50 dilution buffer. All tissue sections were examined and scored by two investigators. Masson’s Trichrome staining was used to detect the interstitial collagen fibers indicative of renal fibrosis.

### 5.11. Statistical Analysis

All data are presented as the means ± SE of three independent experiments. The GraphPad Prism 4.0 and SPSS 12.0 software were analyzing one-way ANOVA and Student’s *t*-test, and were performed to determine significant differences, and *p* < 0.05 or *p* < 0.01 was considered statistically significant.
